# Patterns of Postoperative Trismus Following Mandibulectomy and Fibula Free Flap Reconstruction

**DOI:** 10.3390/cancers15020536

**Published:** 2023-01-16

**Authors:** Rex H. Lee, Cara Evans, Joey Laus, Cristina Sanchez, Katherine C. Wai, P. Daniel Knott, Rahul Seth, Ivan H. El-Sayed, Jonathan R. George, William R. Ryan, Chase M. Heaton, Andrea M. Park, Patrick K. Ha

**Affiliations:** 1 Department of Otolaryngology–Head and Neck Surgery, University of California San Francisco, San Francisco, CA 94158, USA; 2 Department of Oral and Maxillofacial Surgery, University of California San Francisco, San Francisco, CA 94143, USA

**Keywords:** trismus, mouth opening, mandibulectomy, fibula free flap, postoperative, ramus, MIO, interincisal opening, head and neck, survivorship

## Abstract

**Simple Summary:**

Trismus is a serious sequela of head and neck cancer (HNC) treatment that can profoundly affect quality of life. While the relationship between radiotherapy and trismus in HNC has been established, the surgical risk factors for trismus in HNC patients are largely unclear. This study reports the prevalence of postoperative trismus in a large cohort of patients who underwent mandibulectomy and fibula free flap reconstruction. Patients with a posterior mandibulotomy that involved or removed the ramus had significantly higher rates of persistent trismus >6 months after surgery, which was also demonstrated in a multivariable logistic regression. These findings may inform future surgical planning and potentially optimize functional outcomes in patients undergoing significant mandibular resection.

**Abstract:**

The factors that contribute to postoperative trismus after mandibulectomy and fibula free flap reconstruction (FFFR) are undefined. We retrospectively assessed postoperative trismus (defined as a maximum interincisal opening ≤35 mm) in 106 patients undergoing mandibulectomy with FFFR, employing logistic regression to identify risk factors associated with this sequela. The surgical indication was primary ablation in 64%, salvage for recurrence in 24%, and osteonecrosis in 12%. Forty-five percent of patients had existing preoperative trismus, and 58% of patients received adjuvant radiation/chemoradiation following surgery. The overall rates of postoperative trismus were 76% in the early postoperative period (≤3 months after surgery) and 67% in the late postoperative period (>6 months after surgery). Late postoperative trismus occurred more frequently in patients with ramus-involving vs. ramus-preserving posterior mandibulotomies (82% vs. 46%, *p* = 0.004). A ramus-involving mandibulotomy was the only variable significantly associated with trismus >6 months postoperatively on multivariable logistic regression (OR, 7.94; 95% CI, 1.85–33.97; *p* = 0.005). This work demonstrates that trismus is common after mandibulectomy and FFFR, and suggests that posterior mandibulotomies that involve or remove the ramus may predispose to a higher risk of persistent postoperative trismus.

## 1. Introduction

Trismus, or restricted mouth opening, is an increasingly recognized condition among patients with head and neck cancer (HNC). The impact of trismus on quality of life can be devastating, including marked limitations in communication, inadequate nutrition, and chronic pain, which predispose to social isolation and depression [1,2,3]. Difficulty performing adequate dental care with decreased mouth opening also frequently leads to poor oral hygiene and caries, which is especially concerning in irradiated patients given the risk of osteoradionecrosis if dental extraction is required [4]. The prevalence of trismus in HNC patients varies widely by study, from less than 10% to greater than 50% [5,6,7,8]. This broad range suggests that trismus development is influenced by many overlapping demographic and treatment parameters, which are challenging to individually delineate in varied patient cohorts. Furthermore, there is inconsistency in the definition of trismus between HNC care providers, such as the use of subjective jaw mobility assessments rather than quantitative measurements and disagreement over the numerical cutoffs for mouth opening that constitute trismus [6]. These limitations in the literature create barriers to comparing the scope of this problem across patients and institutions.

While radiotherapy (RT) has a well-recognized and dose-dependent relationship with trismus in HNC patients, the influence of surgical interventions on trismus is less clear [8,9,10]. Previous work has demonstrated that certain intraoperative actions may decrease trismus risk, such as prophylactic coronoidectomy, division of the ipsilateral masseter and/or medial pterygoid muscles, and immediate rather than delayed reconstruction of surgical defects [8,11,12,13]. It is essential to identify the surgical factors that predispose to trismus, and understand how these operative features are affected by preoperative clinical characteristics, so that surgical resections may be modified to optimize functional outcomes.

One surgical cohort that may be especially susceptible to postoperative trismus are patients undergoing mandibulectomy with fibula free flap reconstruction (FFFR). These patients represent a unique challenge in the early postoperative period, as physicians may be reticent to initiate early jaw stretching exercises out of concern for stressing the newly placed bone graft. There is little data on the prevalence and severity of trismus in patients with significant mandibular resection (i.e., segmental mandibulectomy resulting in a bony continuity defect). A previous study in HNC patients receiving free flap reconstruction of lateral segmental mandibular defects found that 94% of patients demonstrated “little to no trismus” postoperatively, with a mean follow-up time of 17 months after surgery [14]. In stark contrast, another group reported that approximately 30% of patients experienced trismus following flap reconstruction of posterior mandibular defects at the most recent follow-up (average of 42 months) [15].

Multiple key questions remain unanswered with respect to trismus after mandibulectomy and FFFR. The lack of quantitative mouth opening measurements over time, including change from preoperative baseline to postoperative follow-ups, is a major limitation in determining the natural course and trajectory of this complication. Furthermore, key modifying characteristics that predispose to postoperative trismus following mandibulectomy and FFFR have not been explored, including patients’ preoperative mouth opening, indication for surgery, and anatomy of the bony resection. Identifying these factors may have actionable implications for clinical practice. In this study, we sought to define the scope of trismus in patients after mandibulectomy and FFFR, including factors predictive of worse trismus outcomes.

## 2. Materials and Methods

### 2.1. Patient Selection

The study subjects were identified from a database composed of 277 patients who underwent fibula free flap reconstruction (FFFR) from August 2011 to March 2022. A retrospective chart review of all patients in the database was first performed to determine the patients’ baseline (preoperative) mouth opening status. The patients with a documented preoperative quantitative measurement, most commonly maximum interincisal opening (MIO), were advanced to the next step of the workflow. Aside from MIO, the charts were also queried for multiple related terms indicative of this metric, including “maximum jaw opening” (MJO) and “mouth opening”, as well as units of measurement (“mm” and “cm”). For patients without any quantitative indication of preoperative mouth opening, the otolaryngology provider notes were then searched for a qualitative description of whether the patient had “trismus” or “no trismus” prior to surgery. If available, the degree of mouth opening described in our institution’s preoperative anesthesia note was also used to confirm the trismus status in these patients, with “poor” mouth opening indicating trismus and “good” or “excellent” indicating lack of trismus. An anesthesia mouth opening descriptor of “fair” was considered ambiguous, and such patients were not advanced further if this was the only indication of preoperative trismus status. The patients with neither a preoperative quantitative measurement (referred to collectively as “MIO” from this point forward) nor qualitative description of mouth opening were not included in subsequent steps.

Next, the patient charts that met the above criteria for indication of preoperative mouth opening were searched for quantitative postoperative mouth opening measurements using the same search strategy described above. All documented postoperative MIO measurements were recorded, often from multiple sources, including progress notes from speech–language pathologists and dental oncology colleagues, who work closely with the Head and Neck Surgery program at our institution. All patients with preoperative mouth opening status (either MIO or qualitative) and at least one documented postoperative MIO (*n* = 131) were advanced to the next step of the workflow. For patients without any documented postoperative MIO, the date of the last follow-up with our department was recorded. Those patients without any postoperative MIO and a last follow-up of more than three years prior to the chart review were excluded from further analysis.

The 131 patients meeting the aforementioned preoperative and postoperative measurement criteria then underwent chart abstraction for demographic, clinical, and treatment-related variables. Only the patients who underwent FFFR for segmental mandibulectomy defects (including hemimandibulectomy) were analyzed; 22 patients who had received a maxillectomy prior to FFFR were excluded, as well as 3 patients who received reconstruction only for a remote ablative surgery. This led to a final study size of 106, 52 of whom had both preoperative and postoperative MIO and 54 who had postoperative MIO and only qualitative preoperative mouth opening descriptors.

### 2.2. Defining Trismus and Postoperative Analysis Intervals

We defined trismus as an MIO ≤ 35 mm and the lack of trismus as an MIO > 35 mm, based on multiple prior studies assessing functional and quality of life outcomes [16,17]. The presence or absence of trismus was analyzed at two main intervals: an early postoperative period (≤3 months after surgery) and a late postoperative period (>6 months after surgery). The ≤3 month timepoint was chosen to maximize the number of patients with an early postoperative measurement, as the majority of patients (76/106) had one or two MIOs within this time interval. The late timepoint was intended to encompass persistent postoperative trismus with a higher likelihood of representing a chronic condition; a period of >6 months postoperatively captured at least one MIO in 58/106 patients. In contrast to these early and late timepoints, relatively fewer patients (38/106) had MIOs in the intermediate timepoint between 3 and 6 months after surgery, which also plausibly represents a transition period between acute and chronic trismus in which patients may experience resolution. For patients with multiple MIO measurements within the early or late time periods, the most recent MIO was used. We defined ∆MIO as the difference between a patient’s preoperative MIO and the MIO measured at the most recent follow-up.

### 2.3. Categorizing Mandibulotomy Anatomy

For all patients, the sites of the anterior and posterior mandibulotomies were recorded and broadly divided into “ramus-involving” and “ramus-preserving” cuts. “Ramus-involving” cuts were defined as mandibulotomies that removed any part of the ascending ramus; these cuts were either entirely superior and posterior to the mandibular angle, or they began at the angle and traversed superiorly/posteriorly to involve a portion of the ascending ramus (e.g., spanning from the angle to the sigmoid notch or coronoid process). In contrast, “ramus-preserving” mandibulotomies involved only the angle region itself or were positioned anterior to the angle.

### 2.4. Statistical Analysis

The rates of postoperative trismus at early and late timepoints were compared using the Pearson chi-square test. The proportion of patients with postoperative trismus were compared by surgical indication, presence vs. absence of preoperative trismus, receipt of adjuvant RT/CRT vs. no adjuvant therapy, and ramus-involving vs. ramus-preserving posterior mandibulotomy. We used the Bonferroni correction method to account for multiple comparisons of the preoperative and postoperative trismus rates, with a corrected significance threshold of α < 0.005 (α_original_ of 0.05/11 total comparisons).

Logistic regression was performed with the binary outcome of yes/no trismus >6 months postoperatively, using the most recent MIO available. Univariate logistic regressions were conducted for age, preoperative trismus status, surgical indication, adjuvant therapy, and posterior mandibulotomy location. A multivariable analysis was also conducted with the same variables. Tumor T stage was not included as a variable in the final model, as staging information was available only for patients undergoing primary ablation and not surgery for recurrent disease or osteonecrosis.

The distribution of ∆MIO between groups was visualized with a waterfall plot for all patients with a measurement at >6 months, separated by the same variables used in the regression analyses. To compare the magnitude of ∆MIO change between these variables, the mean ∆MIO between groups were compared via two-tailed student’s *t*-test. Statistical analyses were performed using Stata software version 17.0 (StataCorp LLC; College Station, TX, USA), and the figures were generated via GraphPad Prism 9.3.1.

## 3. Results

### 3.1. Demographic and Treatment Characteristics of the Patient Cohort

A total of 106 patients who underwent mandibulectomy and FFFR met the study criteria. The preoperative demographic and clinical characteristics are presented in Table 1. The cohort was 58% male and 42% female, with an average age of 62.1 years (SD 14.3). The indication for surgery was primary ablation with curative intent in 64% (68/106), salvage resection for recurrent disease in 24% (25/106), and mandibular osteonecrosis in 12% (13/106, including 11 patients with osteoradionecrosis and 2 patients with bisphosphonate-related osteonecrosis of the jaw). Overall, 27% (29/106) of the cohort had a history of prior head and neck irradiation, either for a tumor that subsequently recurred or for a distinct primary tumor prior to developing a second malignancy. Of the patients undergoing surgery for primary ablation with tumor staging available (63/68), the T stage was 11% T1 (7/63), 8% T2 (5/63), 2% T3 (1/63), and 79% T4 (50/63); the N stage was N0 in 49% (31/63) and N+ in 51% (32/63).

Prior to surgery, 45% (48/106) of patients had preoperative trismus, while 55% (58/106) did not have preoperative trismus (Table 1). The posterior mandibulotomy was ramus-involving in 56% (59/106) and ramus-preserving in 44% (47/106). Following mandibulectomy and FFFR, 36% (38/106) received adjuvant radiation (RT), 23% (24/106) received adjuvant chemoradiation (CRT), and 42% (44/106) were not administered adjuvant therapy (Table 1). The proportion of patients administered adjuvant therapy was higher in those without preoperative trismus compared to patients with preoperative trismus (68% vs. 32%, *p* = 0.001) (Table 2). Of those who underwent adjuvant RT/CRT with precise start and end dates available, the median time from surgery to completion of the adjuvant therapy was 112 days (Q_1_ = 99 days, Q_3_ = 131 days). Among all patients, the median time from surgery to the most recent follow-up with our department was 687 days (Q_1_ = 241 days, Q_3_ = 1274 days).

### 3.2. Trismus Prevalence at Early and Late Postoperative Timepoints

In the early postoperative period (≤3 months after surgery), a total of 76 patients had at least one MIO measurement, and the majority experienced trismus (58/76, 76%). Eighty-nine percent of the patients with preoperative trismus demonstrated early postoperative trismus, and 65% of the patients without preoperative trismus demonstrated early postoperative trismus (*p* = 0.014). There were no significant differences in the rates of early postoperative trismus by surgical indication, receipt of adjuvant RT/CRT, or between patients with ramus-involving and ramus-preserving posterior mandibulotomies (Table 2).

In the late postoperative period (>6 months after surgery), a total of 58 patients had at least one MIO measurement. Overall, 67% of patients (39/58) had trismus at this time point. The rates of late postoperative trismus for patients with preoperative trismus and patients without preoperative trismus were 80% and 58%, respectively (*p* = 0.072). Trismus prevalence was again not significantly different when compared by surgical indication or receipt of adjuvant RT/CRT. However, patients with ramus-involving mandibulotomies had significantly higher rates of long-term trismus when compared to ramus-preserving mandibulotomies (82% vs. 46%, *p* = 0.004) (Table 2).

### 3.3. Exploring Variables Associated with Persistent Postoperative Trismus

To delineate the patient-level and treatment-level factors associated with persistent trismus, we employed logistic regression for patients with at least one MIO measurement >6 months postoperatively. For the univariate analysis, the location of the posterior mandibulotomy was the only variable significantly associated with the presence of postoperative trismus at >6 months, with ramus-involving mandibulotomies demonstrating an odds ratio (OR) of 5.52 (95% CI, 1.67–18.17) compared to ramus-preserving mandibulotomies (*p* = 0.005). The presence of existing preoperative trismus approached significance (OR, 2.95; 95% CI, 0.89–9.77; *p* = 0.077). Patients who underwent surgery for osteonecrosis could not be included in the regression model, as all eight osteonecrosis patients with MIO measurements taken >6 months had postoperative trismus (i.e., an indication of osteonecrosis perfectly predicted the regression outcome). This necessitated the reduction of the surgical indication variable from three categories to two (primary ablation vs. salvage for recurrence). Surgical indication, age, and receipt of adjuvant therapy were not significantly associated with persistent postoperative trismus in the univariate analysis (Table 3).

In the multivariable logistic regression with the same variables, posterior mandibulotomy location remained the only variable significantly associated with trismus >6 months postoperatively, with an OR of 7.94 for ramus-involving vs. ramus-preserving cuts (95% CI, 1.85–33.97; *p* = 0.005). As with the univariate analysis, age, surgical indication, and adjuvant therapy were not significantly associated with persistent postoperative trismus in the multivariable regression (Table 3).

### 3.4. Comparing ∆MIO at the Late Postoperative Timepoint

**Figure 1:**
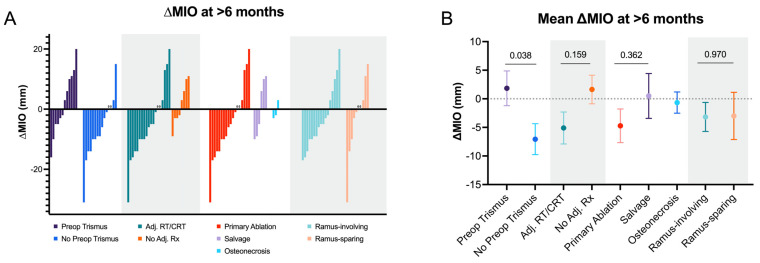
(**A**) Waterfall plot of ∆MIO for all patients with preoperative MIO and MIO measurement taken >6 months postoperatively (*n* = 27), separated by preoperative trismus status, receipt of adjuvant therapy, surgical indication, and ramus involvement of the posterior mandibulotomy; (**B**) mean ∆MIO > 6 months postoperatively for the same 27 patients when compared by preoperative trismus status, receipt of adjuvant therapy, surgical indication, and ramus involvement of the posterior mandibulotomy.

Of the 58 total patients with postoperative MIO measurements taken >6 months postoperatively, 27 patients also had a preoperative MIO available. The overall distribution of ∆MIOs from the preoperative visit to the most recent visit’s MIO measurement >6 months postoperatively for these 27 patients is shown in Figure 1A, separately stratified by preoperative trismus status, receipt of adjuvant therapy, surgical indication, and ramus involvement of the posterior mandibulotomy. The mean ∆MIOs between these groups at the same timepoint are displayed in Figure 1B. On average, patients without preoperative trismus had a decline in ∆MIO, while those with existing preoperative trismus had a mean positive change in ∆MIO (−7.07 vs. +1.83 mm, *p* = 0.038). The mean ∆MIO for patients who received adjuvant RT/CRT was −5.11 mm and +1.63 mm in those without adjuvant therapy (*p* = 0.159). By indication, the mean ∆MIOs for primary ablation, salvage, and osteonecrosis were −4.72, +0.50, and −0.67 mm, respectively (*p* = 0.362 for a comparison between primary ablation and salvage surgery). The mean ∆MIO for the ramus-involving vs. ramus preserving mandibulectomies were −3.18 and −3.00 mm, respectively (*p* = 0.970).

## 4. Discussion

Trismus is a complex, multifactorial condition that represents a formidable challenge in patients with HNC. In surgically treated patients, data on trismus outcomes are limited, especially when compared to the preponderance of work assessing the relationship between trismus and RT. Many previous studies utilize heterogenous HNC patient cohorts receiving a variety of treatment approaches, which makes it difficult to disentangle the individual demographic and treatment-related characteristics that contribute to this sequela. In this study, we focused specifically on one unique surgical population—those undergoing mandibulectomy and FFFR.

The first step towards conducting meaningful trismus studies that are generalizable across patients and institutions is specifying an appropriate definition of trismus. The current evidence suggests that a mouth opening of ≤35 mm is a suitable and clinically meaningful demarcation of trismus that predicts health-related quality of life [16,17]. However, even in the presence of an appropriate trismus metric, obtaining consistent post-treatment jaw opening measurements is a significant challenge. By far, the largest obstacle in this study was a lack of regular MIO measurements taken at both preoperative and postoperative visits. From a database of 277 fibula free flap patients, fewer than half (131/277) had postoperative MIO measurements and either a quantitative or qualitative indication of preoperative mouth opening. Furthermore, of the 106 patients who met the final study criteria, only 52 had a quantitative mouth opening measurement (MIO) taken preoperatively.

Because most patients in this cohort did not have both baseline preoperative MIOs and regular postoperative MIOs, our ability to compare the magnitude of change in mouth opening over time was limited (only 27 total patients with follow-up >6 months after surgery had both preoperative and postoperative MIOs). Nonetheless, the comparison between the mean ∆MIO at >6 months was significant between the patients with preoperative trismus compared to the patients without preoperative trismus (∆MIO_avg_ of +1.83 vs. −7.07 mm, respectively; *p* = 0.038). Indeed, while half (6/12) of the patients with preoperative trismus experienced increased MIO at >6 months, only 13% (2/15) of patients without preoperative trismus demonstrated an increase in MIO at this late time point. This suggests that while patients who are trismus-free preoperatively will generally experience a decline in postoperative MIO, patients with existing preoperative trismus may exhibit a marginal improvement in postoperative MIO. However, it is important to recognize that the majority of patients with preoperative trismus continued to have chronic trismus (i.e., MIO ≤ 35 mm) postoperatively. In addition, because MIO measurements were taken at different intervals for different patients, it was not feasible to infer a general timeline of the trajectory of MIO decline following mandibulectomy and FFFR. Future work that incorporates consistent mouth opening measurements at defined and frequent postoperative intervals may allow for the construction of a generalizable timeline for the development of (and recovery from) trismus in this population.

A key limitation of this study was our inability to assess the impact of jaw stretching interventions on the risk of trismus development in this cohort of mandibulectomy and FFFR patients. At our institution, home jaw stretching and neck stretching exercises are routinely taught and implemented with postsurgical patients by our team of speech–language pathologists (SLPs). However, details on the adherence to these regimens and the time periods over which stretching is practiced are often unable to be accurately extrapolated from retrospective chart review. The limitations of the documentation also precluded the analysis of the benefit of active device-based intervention (using products such as the TheraBite^®^ or OraStretch^®^) in this study. Given the high out-of-pocket cost of these devices coupled with inconsistent clearance by insurance providers, it is often unclear if patients even received the stretching device recommended to them, let alone the adherence to the stretching exercises with the device itself. In patients with mandibulectomy and FFFR, concern for imparting excessive stress on the newly placed vascularized bone graft may generate reticence over initiating early jaw stretching exercises postoperatively. There have been reports of serious complications while using the TheraBite^®^ in the post-treatment setting, including a mandibular fracture in an HNC patient with undiagnosed mandibular osteoradionecrosis (ORN) [18], and fracture of titanium mandibular reconstruction plates in the setting of mandibular recurrence following mandibulectomy and FFFR [19]. However, it is notable that both complications occurred in patients with bone that was ostensibly already structurally compromised (due to ORN or recurrent cancer). The true risk of trismus devices in mandibulectomy and FFFR patients with a healthy flap and expected postoperative healing has not been studied. Additional work will help to clarify the earliest time period following surgery that is safe for device-assisted jaw stretching, and the optimal timing for trismus interventions in these patients.

Our work suggests that the location of the posterior mandibulotomy may affect the risk of postoperative trismus after mandibulectomy and FFFR. At >6 months, patients with ramus-involving posterior mandibulotomies experienced trismus at nearly twice the rate of patients with ramus-preserving cuts (82% vs. 46%), the most robust difference in magnitude among any variables compared in this study. Multivariable logistic regression revealed a nearly eight-fold greater odds of persistent postoperative trismus for patients with ramus-involving posterior mandibulotomies, when adjusted for age, preoperative trismus status, surgical indication, and receipt of adjuvant therapy. However, we could not demonstrate a significant difference in ∆MIO between these groups, most likely due to the very small number of patients with paired preoperative and postoperative MIO measurements, as discussed above. There are multiple possible explanations for the observation of marked differences in late postoperative trismus rates by mandibulotomy location. Acute or chronic inflammation of structures surrounding the mandibular ramus, condyle, or coronoid can either directly limit movement around the temporomandibular joint and/or induce pain resulting in a reflex trismus (as is often also seen in non-neoplastic conditions such as peritonsillar abscesses or lateral pharyngeal space infections) [20]. Violation of the region surrounding the ramus during surgery may also result in the fibrotic shortening of the pterygoids or pterygomandibular ligament during healing, thereby further contracting mouth opening. While the extent of disease is the largest factor dictating whether the ramus can be spared during resection, our data identify patients at a particularly high risk of late postoperative trismus, who may especially benefit from vigilant surveillance of mouth opening and early trismus interventions. It is also critical to note that a large portion of HNC patients undergoing mandibulectomy and FFFR will demonstrate adverse features on surgical pathology, necessitating adjuvant RT/CRT for optimal oncologic control. Radiation itself has a well-known, dose-dependent relationship with trismus secondary to fibrosis of the masticatory apparatus, especially with large doses to the masseter and medial pterygoid muscles [21,22,23]. It will be important to determine how radiation interacts with postoperative anatomy in mandibulectomy and FFFR patients, and how irradiation of the retained masticatory musculature contributes to trismus severity specifically following surgical disruption of the ramus.

## 5. Conclusions

In summary, this study described the scope of postoperative trismus following mandibulectomy and FFFR, including potential contributing factors to this sequela. We demonstrated that most patients (76%) experienced trismus in the early (≤3 months) postoperative period and persistent trismus remained common in the late (>6 months) postoperative period (occurring in 67% of patients in this study). Using logistic regression, we found that a posterior mandibulotomy involving or removing the ramus was associated with a substantially higher odds of persistent trismus after surgery. To our knowledge, this is the first study that implicates mandibulotomy location as a surgical risk factor for postoperative trismus. Larger-scale studies are critical for identifying patients at the highest risk of trismus after mandibulectomy with FFFR, and delineating precise temporal changes in mouth opening may inform key postoperative intervals for active trismus intervention.

## Figures and Tables

**Table 1:** Baseline demographic characteristics and treatment variables for the study cohort.

		*n* (%)
Age	Mean (SD)	62.1 (14.3)
Sex	Male	62 (58)
	Female	44 (42)
Surgical Indication	Primary Ablation	68 (64)
	Salvage	25 (24)
	Osteonecrosis	13 (12)
Stage (primary tumors)	T1/T2	12 (11)
	T3/T4	51 (48)
	N0	31 (29)
	N+	32 (30)
	Stage NA/NR	43 (41)
Preoperative Trismus	Present	48 (45)
	Absent	58 (55)
Adjuvant Therapy	Adjuvant RT/CRT	62 (58)
	None	44 (42)
Posterior Mandibulotomy	Ramus-Involving	59 (56)
	Ramus-Preserving	47 (44)

SD, standard deviation; RT, radiation; CRT, chemoradiation; NA, not available; NR, not relevant (for indications of salvage or osteonecrosis).

**Table 2:** Proportion of patients experiencing trismus at three timepoints: preoperative baseline, early postoperative period (≤3 months after surgery), and late postoperative period (>6 months after surgery).

		Preop. Baseline (N = 106)			≤3 Months Postop. (N = 76)			>6 Months Postop. (N = 58)		
		Trismus *n* (%) (*N* = 48)	No Trismus *n* (%) (*N* = 58)	*p*-Value	Trismus *n* (%) (*N* = 58)	No Trismus *n* (%) (*N* = 18)	*p*-Value	Trismus *n* (%) (*N* = 39)	No Trismus *n* (%) (*N* = 19)	*p*-Value
Surgical Indication	Primary Ablation	24 (35)	44 (65)	0.003 *	34 (71)	14 (29)	0.313	21 (58)	15 (42)	0.070
	Salvage	13 (52)	12 (48)		15 (83)	3 (17)		10 (71)	4 (29)	
	Osteonecrosis	11 (85)	2 (15)		9 (90)	1 (10)		8 (100)	0 (0)	
Preoperative Trismus	Present	48 (100)	-	-	32 (89)	4 (11)	0.014	20 (80)	5 (20)	0.072
	Absent	-	58 (100)		26 (65)	14 (35)		19 (58)	14 (42)	
Adjuvant Therapy	Adjuvant RT/CRT	20 (32)	42 (68)	0.001 *	31 (74)	11 (26)	0.568	24 (62)	15 (38)	0.185
	None	28 (64)	16 (36)		27 (79)	7 (21)		15 (79)	4 (21)	
Posterior Mandibulotomy	Ramus-Involving	33 (56)	26 (44)	0.014	34 (81)	8 (19)	0.291	28 (82)	6 (18)	0.004 *
	Ramus-Preserving	15 (32)	32 (68)		24 (71)	10 (29)		11 (46)	13 (54)	

Preop., preoperative; Postop., postoperative; RT, radiation; CRT, chemoradiation. Asterisks (*) designate significance with α < 0.005 (Bonferroni correction for multiple comparisons).

**Table 3:** Univariate and multivariable logistic regressions for the presence of persistent late postoperative trismus (>6 months postoperatively).

		Univariate Model		Multivariable Model	
		OR (95% CI)	*p*-Value	OR (95% CI)	*p*-Value
	Age	1.00 (0.96–1.05)	0.876	1.00 (0.95–1.04)	0.906
Preoperative Trismus	Absent	Ref.		Ref.	
	Present	2.95 (0.89–9.77)	0.077	0.81 (0.16–4.17)	0.799
Surgical Indication	Primary Ablation	Ref.		Ref.	
	Salvage	1.79 (0.47–6.79)	0.395	2.00 (0.29–13.59)	0.478
	Osteonecrosis	-	-	-	-
Adjuvant Therapy	None	Ref.		Ref.	
	Adjuvant RT/CRT	0.43 (0.12–1.53)	0.191	1.10 (0.13–9.58)	0.933
Posterior Mandibulotomy	Ramus-Preserving	Ref.		Ref.	
	Ramus-Involving	5.52 (1.67–18.17)	0.005	7.94 (1.85–33.97)	0.005

RT, radiation; CRT, chemoradiation.

## Data Availability

De-identified data presented in this study are available upon reasonable request from the corresponding author.

## Abbreviations

FFFR	Fibula free flap reconstruction
HNC	Head and neck cancer
MIO	Maximum interincisal opening
OR	Odds radio
Q_1_	First quartile
Q_3_	Third quartile
RT/CRT	Radiation/chemoradiation
SD	Standard deviation
SLP	Speech–language pathologist
